# Fabrication of an Fe-Doped ZIF-67 Derived Magnetic Fe/Co/C Composite for Effective Removal of Congo Red

**DOI:** 10.3390/molecules29092078

**Published:** 2024-04-30

**Authors:** Yu Cao, Zeming Dai, Xuan Zhou, Yuting Lin, Jianhua Hou

**Affiliations:** 1 College of Environmental Science and Engineering, Yangzhou University, Yangzhou 225000, China; dzmzeming@163.com (Z.D.); yzdxzx@yeah.net (X.Z.); linyut@yeah.net (Y.L.); 2 Jiangsu Collaborative Innovation Center for Solid Organic Waste Resource Utilization, Nanjing 210095, China

**Keywords:** dye absorption, Fe-doped MOF, Congo red, magnetic material, Fe/Co/C composite

## Abstract

The dyes in printing and dyeing wastewater are harmful to the human body and the environment. It is essential to develop practical and effective adsorbents to deal with them. In this study, an Fe-doped, ZIF-67 derived Fe/Co/C composite material with strong magnetism was successfully synthesized. The effects of pH, initial concentration, and adsorption time on the properties of the adsorbent were investigated. To further improve the removal efficiency and enhance the practicality, potassium peroxymonosulfate (PMS) was added to the system due to its Fenton-like effect. Then, an Fe/Co/C composite was used with PMS to remove Congo red (CR) with a 98% removal of 250 mg·L^−1^. Moreover, for its high saturation magnetization of 85.4 emu·g^−1^, the Fe/Co/C composite can be easily recovered by applying a magnetic field, solving the problem that powdery functional materials are difficult to recover and, thus, avoiding secondary pollution. Furthermore, since the composite material was doped before carbonization, this synthetic strategy is flexible and the required metal elements can be added at will to achieve different purposes. This study demonstrates that this Fe-doped, ZIF-67 derived magnetic material has potential application prospects for dye adsorption.

## 1. Introduction

With the development of industrialization, the pollution of printing and dyeing wastewater is becoming more and more serious. Among them, dyes are often used in textile, coating, plastic, and paper industry. Usually, many dyes are toxic. The discharge of these toxic dye sewages will cause harm to human health and the ecosystem [1,2]. In particular, the anionic dye Congo red (CR) has carcinogenic and mutagenic effects on human health [3,4]. Thus, dye treatment methods with low-cost and high-efficiency must be developed. At present, the main treatment strategies of dye wastewater are photocatalysis [5,6] and adsorption [7,8], and the adsorption method is widely used for its characteristics of being efficient and cheap [9,10]. So far, various traditional adsorbents such as zeolite [11,12], chitosan [13,14], montmorillonite [15,16], and biochar [17,18,19] have been reported for dye adsorption. However, poor adsorption capacity, low reuse rate, and high cost limit their further application [20,21] Consequently, the development of novel adsorbents with high adsorption performance, low production price, and recyclability presents great opportunities and challenges.

Metal–organic frameworks (MOFs) are crystalline porous materials with periodic network structures, which were formed via the self-assembly of organic ligands and metal coordination centers (metal ions or metal clusters) [22,23,24]. As new functional materials, MOFs have the advantages of high specific surface area, fixed porosity, and various topological structures [25,26,27]. At present, many studies have revealed that MOF or MOF based composites have potential applications for the treatment of dyes in wastewater [28,29]. Sharma et al. summarized a recent study of advanced oxidation processes using MOF materials for water depollution [30]. In this review, the authors listed various MOFs and their hybrid materials for dyes degradation. Mahmoodi et al. reported a series of ZIF-8/GO/CNT hybrid nanocomposites for malachite green (MG) removal [31]. The prepared materials showed outstanding adsorption capacity. In addition, these materials were stable and had high reusability after four cycles. Arora et al. developed an Fe-BDC MOF for methylene blue (MB) removal from industrial wastewater [32]. The results of the study revealed that 94.74% of MB could be removed within 24 h with the use of 25 mg Fe-based MOF. The mechanism study indicated this adsorption process was mainly chemical adsorption. Hou’s group constructed a cage-like Zn-MOF with fcu topological structure [33]. The unique structure of this novel MOF made it display a variety of functions, such as gas adsorption/separation and CO_2_ conversion. In addition, this Zn-MOF had potential application for selective adsorption of organic dye MB. Recently, Lee et al. systematically summarized the research work of dye removal from wastewater using MOFs [34]. They discussed the characterization of dyes, how to remove the dyes, the factors affecting dye adsorption, the fabrication and modification of MOF, and the future development of this field.

However, MOF usually exists in the form of crystalline (micron level) or powder (nanoscale). It is difficult to recycle when applied in the real environment [35,36]. Some of the metal ions in MOFs are harmful to the environment. The development of MOF-based materials that are easy to recycle and are non-toxic is highly desirable. Herein, we propose an Fe-doped, ZIF-67 derived magnetic nanomaterial and it was employed to remove CR. The Fe/Co/C material is friendly to the environment and can be easily recovered due to its strong magnetic property. This MOF-based environmental functional material is expected to provide solutions and technological innovations for the treatment of pollutants in wastewater.

## 2. Results and Discussion

**Figure 1:**
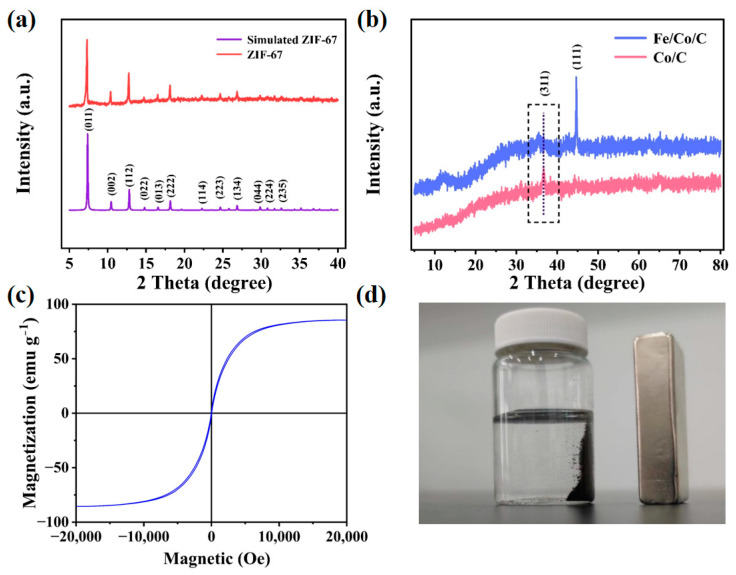
(**a**) XRD patterns of the simulated and as-synthesized ZIF-67, (**b**) XRD patterns of Fe/Co/C and Co/C, (**c**) VSM analysis of Fe/Co/C, and (**d**) magnetic display of the Fe/Co/C material.

Figure 1a presents the XRD pattern of the prepared material. It is clear that all the diffraction peaks of the as-synthesized sample were in good agreement with the simulated XRD pattern of ZIF-67, which indicates that ZIF-67 was successfully synthesized. The XRD pattern for the resulting sample has (011), (002), (112), (022), (013), (222), (114), (233), (134), and (044), which are the characteristic peaks of ZIF-67 [37]. Then, the adsorption properties of carbonized Co/C composite with different ZIF-67 contents were studied (as shown in Figure S2). While using 20 mL CR solution (the concentration was 25 mg·L^−1^) and 10 mg of the prepared material, it was found that when the mass of ZIF-67 accounted to 20%, the material had the best adsorption effect on CR, so the subsequent experiments were all based on this.

To further improve the adsorption performance, FeCl_3_ was added to the colloidal ZIF-67@chitosan material (shown in Figure S3), and the precursor of Fe/Co/C was prepared by stirring. After the same carbonization steps of Co/C, the Fe/Co/C material was obtained. As illustrated by the red curve in Figure 1b, the strong diffraction peak at 36.7° belonged to (311) of Co_3_O_4_ (PDF#73-1701). By comparing the XRD patterns of Co/C and Fe/Co/C, the new strong diffraction peak of Fe/Co/C at 44.6 belongs to (111), and the successful addition of Fe is confirmed by comparing the shift of the peak. It was found that Fe/Co/C had a significantly higher adsorption capacity for CR than Co/C, which is shown in Figure S4. This may be attributed to the addition of the Fe element, which increases the amount of active sites in the material, thus improving the efficiency [32]. According to Formula (1), the crystallite size is 47.1 nm.

As is known to all, the BET surface area and pore size were very important to the adsorption performance. Hence, the Fe/Co/C composite was further revealed via a N_2_ sorption experiment (Figure S5a). The adsorption–desorption isotherm of this material was a combination of type I and type IV, indicating the material contains both microporous and mesoporous pores. Moreover, from the pore size distribution curve (Figure S5b), it can be observed that micropores are the main form of existence in the material (<2 nm), followed by a small part of mesopores. Figure S6a shows the BET analysis of Co/C. Through comparison, it was found that the specific surface area of the carbonized material decreases, which may be caused by the carbonization of chitosan. When comparing the pore size distribution of Co/C (Figure S6b) and Fe/Co/C (Figure S5b), it was found that the pore size of the carbonized materials changed from mesopores to a mixed state of mesoporous and micropores, which meant that the introduction of Fe was conducive to the formation of hierarchical pores, providing favorable conditions for adsorption. Furthermore, the BET surface area of Fe/Co/C is 920.2 m^2^∙g^−1^. Due to this high BET surface area and abundant porous characteristics, this Fe/Co/C material possessed good adsorption ability.

Figure S7 shows the infrared spectra of ZIF-67 and Fe/Co/C materials. It can be seen that ZIF-67 has a peak in Co-N bonds at 424 cm^−1^, while the peak at 670–760 cm^−1^ belongs to C-H bonds and the peak at 990–1420 cm^−1^ belongs to C-N bonds. The peak of 1572 cm^−1^ belongs to C=N and the peak of 1593 cm^−1^ belongs to the C=C bond, because after two high-temperature carbonizations, only the C=C bond of the Fe/Co/C material corresponds to ZIF-67.

In order to facilitate recycling, the magnetic properties of the material were also studied. As shown in Figure 1c, the Fe/Co/C magnetic features obtained from VSM analysis ranged from −20,000 to 20,000 Oe at room temperature. It was shown that the hysteresis loop of the sample has the characteristics of low coercivity and low remanence, which accords with the characteristics of soft magnetic materials [38]. The saturation magnetization of Fe/Co/C is 85.4 emu·g^−1^, indicating that the material has good magnetism and is conducive to recovery.

As revealed in Figure 1d, Fe/Co/C possesses strong magnetic properties, which may be due to the formation of magnetic substances such as Fe_3_O_4_ and Co_3_O_4_. Thus, this material may be suitable for pollutant absorption.

**Figure 2:**
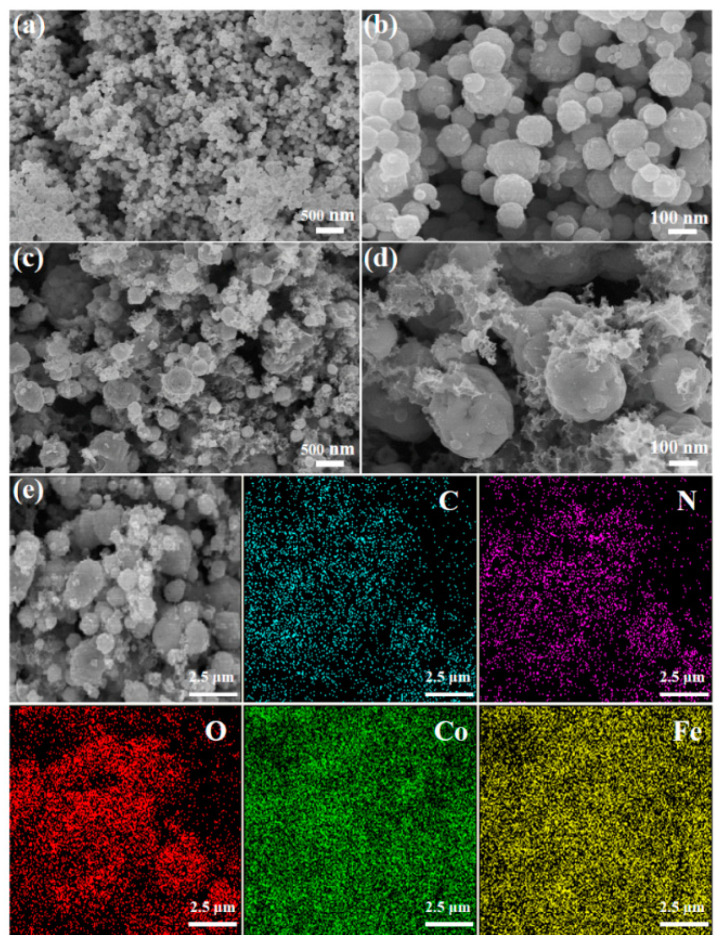
(**a**,**b**) SEM images of the Co/C material, (**c**,**d**) SEM images of the Fe/Co/C material, and (**e**) EDS mapping images of Fe/Co/C material.

To study the morphology of the materials, the SEM analysis was employed. As shown in Figure 2a,b, it is obvious that the Co/C material was comprised mainly of spherical forms. Figure 2c,d provides the SEM photographs of the Fe/Co/C material. Compared with the images of the Co/C material, there is an extra layer of particles on the surface of Fe/Co/C, which should be caused by the addition of FeCl_3_. For qualitative analysis, EDS mapping was carried out. From the EDS mapping images of Fe/Co/C in Figure 2e, it is observed that Fe and Co elements were evenly distributed, which further proves the successful addition of Fe element.

**Figure 3:**
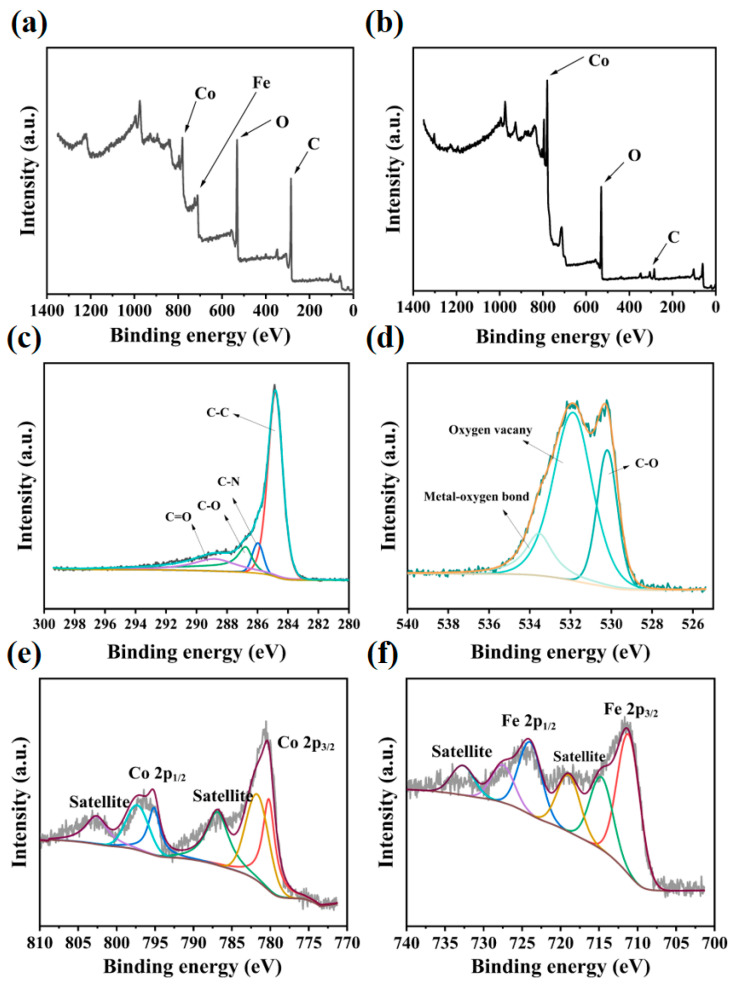
(**a**) XPS spectra of Fe/Co/C material. (**b**) XPS spectra of Co/C material. (**c**) High-resolution XPS spectra of C 1s. (**d**) High-resolution XPS spectra of O 1s. (**e**) High-resolution XPS spectra of Co 2p. (**f**) High-resolution XPS spectra of Fe 2p.

To detect the chemical structure of the above materials, X-ray photoelectron spectroscopy (XPS) was performed. Table 1 shows the proportion of surface elements obtained via XPS. Figure 3a,b show the comparison of XPS scanning images of Co/C and Fe/Co/C materials. The appearance of Fe peaks confirms the successful introduction of Fe elements, while all N elements fall off due to high temperature carbonization. Figure 3c,d shows the C 1s and O 1s XPS peaks of Fe/Co/C materials. The four peaks of C1s are the C-C bond at 284.8 eV, C-N bond at 285.9 eV, C-O bond at 286.8 eV, and C=O bond at 288.8 eV [39]. O 1s has three peaks: a C-O bond at site 530.2 eV, an oxygen vacancy at 531.8 eV, and a metal–oxygen bond at 533.5 eV [40]. As displayed in Figure 3e,f, an additional peak of Fe 2p was found at the 711.0 eV, which confirms the successful addition of the Fe element. Figure 3c,d show the high-resolution spectra of Co 2p and Fe 2p of Fe/Co/C. Usually, Co mainly has four peaks, among which the peak at 781.0 eV of Co 2p_3/2_ belongs to the oxide of Co (Co^3+^/^2+^). The remaining peaks appear at 786.7 eV, corresponding to the satellite peak [41,42]. Fe 2p is mainly divided into three peaks, Fe 2p_3/2_ at 711.8 eV, Fe 2p_1/2_ at 724.8 eV, and satellite peak at 718.4 eV, which were consistent with the peak of Fe_3_O_4_ [36]. 

**Figure 4:**
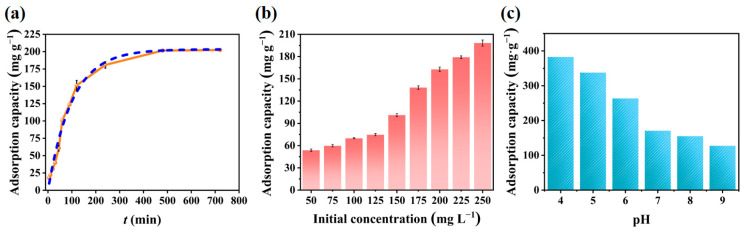
(**a**) Adsorption capacity of Fe/Co/C varies with time. (Blue is the fitted curve) (**b**) Effect of different initial concentration on adsorption capacity. (**c**) The effect of different pH values on adsorption capacity.

The time when the material reached adsorption equilibrium was important, thus it was evaluated via the following experiment. The results are presented in Figure 4a. In the first 180 min, the material rapidly adsorbed CR in water and then the adsorption rate gradually slowed down; the adsorption capacity was 201.6 mg∙g^−1^ at 480 min and 202.4 mg∙g^−1^ at 720 min, respectively. It is clear that the adsorption capacity of the material did not increase significantly from 480 min to 720 min. Therefore, 480 min was selected as the adsorption equilibrium time for CR adsorption.

The effects of CR solution with different initial concentrations (50 mg∙L^−1^~250 mg∙L^−1^) on the adsorption capacity of Fe/Co/C at adsorption equilibrium are shown in Figure 4b. It was found that the initial concentration was significant to the adsorption capacity of the material. With the increase in the initial concentration, the adsorption capacity increased; the adsorption capacity was 180.3 mg∙g^−1^ and 198.7 mg∙g^−1^ at the concentration of 225 mg∙L^−1^ and 250 mg∙L^−1^. In addition, Figure 4b reveals that the improvement in adsorption effect was not obvious when the initial concentration continued to increase, demonstrating that the adsorption was almost saturated.

pH is a significant factor in adsorption experiments, the pH effect on the adsorption capacity of Fe/Co/C for CR was further tested. In this experiment, the pH was adjusted between 4 and 9 (Figure 4c). It was found that the adsorption capacity of CR reached the maximum value of 382.8 mg∙g^−1^ at pH = 4 and then decreased significantly with the increase in pH. This may be attributed to the fact that CR is typically a negatively charged anionic dye in aqueous solution. Furthermore, the surface of Fe/Co/C is positively charged, so it is more conducive for CR adsorption [43].

Figure S8a–c show the Langmuir, Freundlich. and Dubinin–Radushkevich model isotherms of Fe/Co/C composites, and the parameters of the isotherm, in detail, are shown in Table 2. It is evident that the R^2^ of the Langmuir model of Fe/Co/C is closer to 1 than the R^2^ of the Freundlich model and Dubinin–Radushkevich model. Therefore, the Langmuir model can better describe the experimental results. According to the Langmuir isotherm, the maximum adsorption capacity of Co/C on CR is 200.8 mg∙g^−1^.

In summary, the adsorption capacity of the Fe/Co/C material is the highest when the pH is 4, and it takes 720 min to reach the adsorption equilibrium, which is more consistent with the Langmuir model; the maximum adsorption capacity of Congo red Dye is 200.8 mg·g^−1^.

**Scheme 1:**
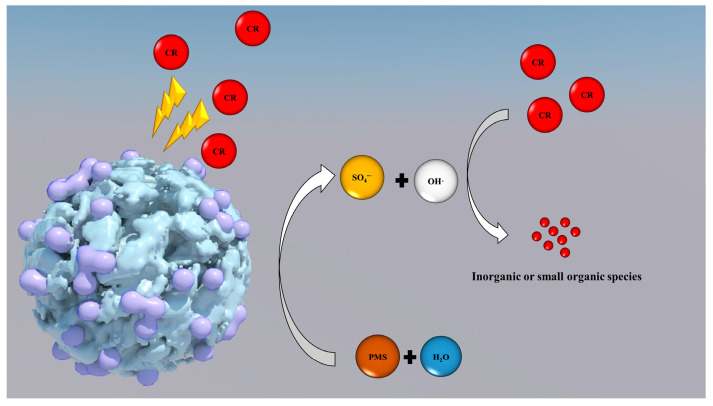
An illustration of the adsorption procedure mechanism.

To study the possible adsorption mechanism, the adsorption kinetics of Fe/Co/C for CR were further studied (shown in Figure S9). The results of experimental data modeling using the pseudo-first-order model and pseudo-second-order model are shown in Table 3. It was demonstrated that the pseudo-second-order kinetic model can better describe the experimental results. Since pseudo-second-order kinetic model is the basis of chemisorption [35,44], the adsorption of CR via Fe/Co/C is attributed to chemisorption. Scheme 1 provides an illustration of the adsorption procedure mechanism.

**Figure 5:**
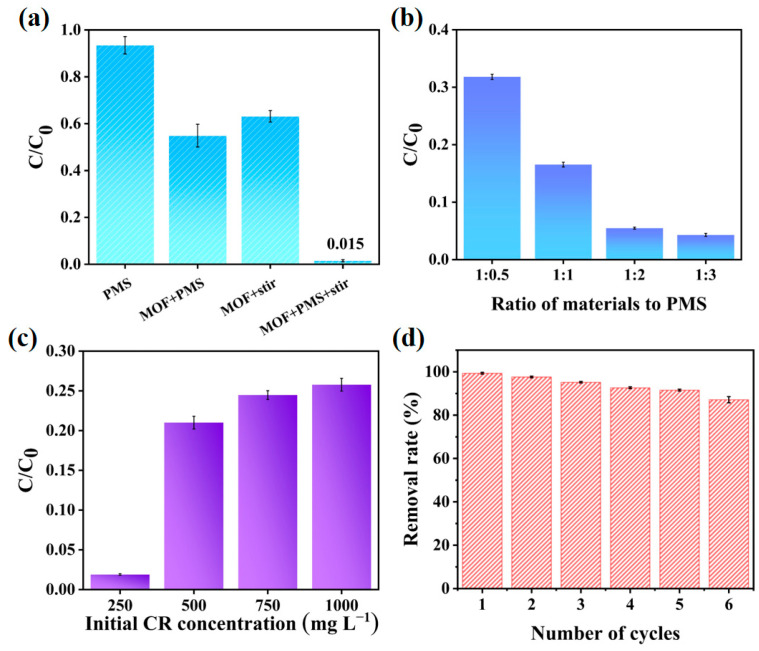
(**a**) The effect of different conditions on the adsorption capacity of the Fe/Co/C material. (**b**) The effect of PMS concentration on the CR removal ability of materials. (**c**) The effect of different initial concentrations of CR removal via PMS and Fe/Co/C systems. (**d**) Recycling experiment diagram of Fe/Co/C material.

In order to further improve the removal efficiency of Fe/Co/C materials for CR and enhance the practicality of the materials, PMS was added to the system due to its Fenton-like effect. By setting different conditions, the effect of PMS on the material for the removal of CR was studied. As shown in Figure 5a, adding PMS itself hardly affects the concentration of CR, indicating that PMS is invalid for CR removal. When both PMS and Fe/Co/C were added, the removal efficiency was effectively improved. Compared with the situation of only adding PMS, the removal efficiency increased by about 48.6%. This phenomenon pointed out that PMS can react with the Fe/Co/C composite. This may be because adding Fe and Co in the material activates the persulfate, which interacts with water to produce sulfate and hydroxyl radicals. Then, the produced sulfate and hydroxyl radicals oxidize the CR in the water [45,46]. Subsequently, it was found that the removal efficiency was greatly improved after adding Fe/Co/C and PMS together under stirring. For 250 ppm CR, the removal rate reached 98%, which was 59.5% higher than that when adding Fe/Co/C and PMS without stirring. This may be caused by the synergistic effect between the adsorption of the material and the catalytic oxidation of free radicals produced by PMS.

The influence of PMS dosage on the removal effect is explored in Figure 5b. The ratio of Fe/Co/C and PMS dosage was set as 1:0.5, 1:1, 1:2, and 1:3, respectively. It was found that with the increase in PMS dosage the removal efficiency became higher, which may have been because more sulfate and hydroxyl radicals were produced with the increase in PMS. However, the differences in the promotion effect between the ratio of 1:2 and 1:3 are not significant, demonstrating that Fe/Co/C has an upper limit on the activation of PMS. Moreover, the initial concentration of CR also impacts Fe/Co/C and PMS systems. As shown in Figure 5c, the removal efficiency also increased with the increase in CR concentration. The main adsorption process of CR via the Fe/Co/C and PMS systems is illustrated in Figure S10.

For adsorption materials, the cycle performance is a very important evaluation factor. In this study, CR at a concentration of 400 mg·L^−1^ was used for the cycling experiments. The amount of Fe/Co/C was 1 g·L^−1^ and the amount of sodium persulfate was 2 g·L^−1^. After one cycle, the materials were recovered using a magnet. The materials were washed several times with deionized water and ethanol and dried in an oven. The results of the cycling experiments are shown in Figure 5d. The results showed that, after six cycles, the processing capacity of the material for CR decreased to 87.1%. The decrease in efficiency may have been due to the adsorption of dyes and reaction intermediates on the surface of the material, which could not be removed via centrifugal washing [47]. This may result in the material not being able to adequately contact the dye and PMS, thus reducing the treatment efficiency.

In order to further evaluate this material, it was compared with other dye-adsorbent magnetic materials reported in recent years Ref [48,49,50,51,52,53]. The results are shown in Table S1. Through comparison, it was found that this Fe/Co/C material has a certain CR dye adsorption capacity and possesses high saturation magnetization. Due to these characteristics, the Fe/Co/C composite can be easily recovered by applying a magnetic field, solving the problem that powdery functional materials are difficult to recover and, thus, avoiding secondary pollution.

## 3. Materials and Methods

### 3.1. Materials

Cobalt nitrate hexahydrate [Co(NO_3_)_2_·6H_2_O], 2-methylimidazole, chitosan, and potassium persulfate (PMS) were purchased from Shanghai Aladdin Biochemical Technology Co., LTD. Methanol, ethanol, acetic acid, hydrogen chloride (HCl), ferric chloride (FeCl_3_), sodium hydroxide (NaOH), and Congo red (CR) were purchased from Sinopharm Chemical Reagent Co., Ltd. (Shanghai, China). All the materials were used as received without further purification.

### 3.2. Methods

X-ray diffraction (XRD) patterns of the materials were recorded using a D/max-2550 diffractometer with Cu Kα radiation (λ = 1.5418 Å) at 50 kV and 200 mA (Rigaku, Japan). The morphologies of the obtained materials were characterized using a S4800 scanning electron microscopy (SEM, HITACHI, Tokyo, Japan). N_2_ adsorption–desorption isotherm was measured at 77 K using ASAP 2460 (Micromeritics, Norcross, GA, USA). The Brunauer–Emmett–Teller (BET) surface area was calculated using the adsorption data at a relative pressure ranging from 0.05 to 0.3. The pore size distributions were estimated via the NLDFT (non-local density functional theory) method. UV testing was carried out with a 2250 UV-visible spectrometer (Shimadzu, Japan).

### 3.3. Synthesis of ZIF-67@chitosan

ZIF-67 was synthesized according to a previous report with slight modifications [54]. In brief, 2 mmol of Co(NO_3_)_2_·6H_2_O was dissolved in 30 mL methanol, and 12 mmol of 2-methylimidazole was dissolved in 10 mL methanol. Then, the 2-methylimidazole solution was added to the Co(NO_3_)_2_·6H_2_O solution slowly via vigorous stirring, resulting in a purple precipitate. After the two solutions were thoroughly mixed, it was kept for 24 h at room temperature. Finally, the purple precipitate was separated via centrifugation and washed with ethanol three times before drying overnight under vacuum at 60 °C.

Different proportions of ZIF-67 and chitosan precursors were prepared here, among which the mass of ZIF-67 was 0, 5, 10, 15, 20, 25, 30, 40, and 50 wt/wt% of chitosan, with 0.25 g of chitosan as the standard.

After that, ZIF-67 and chitosan were dissolved in 25 mL of 2% acetic acid solution and stirred for 3 h. The excess water of the system was evaporated in a water bath (90 °C) and washed with ethanol via centrifugation several times until the ethanol solution was colorless. After drying in the oven, the colloidal ZIF-67@chitosan material was obtained.

### 3.4. Synthesis of ZIF-67/FeCl_3_@chitosan

Based on the above experiment, 0.0347 g of FeCl_3_ was mixed with the colloidal ZIF-67@chitosan material (25 wt/wt%) and they were continuously stirred for another 3 h. The rest of the procedure was the same. Then, the obtained sample was washed with ethanol via centrifugation several times until the ethanol solution was colorless and then it was placed in the oven for drying overnight.

### 3.5. Synthesis of Co/C and Fe/Co/C Material

Firstly, a desired amount of ZIF-67@chitosan and ZIF-67/FeCl_3_@chitosan was weighted and placed into the combustion boat, respectively. Then, the combustion boat was put into the tubular furnace in N_2_ atmosphere. The temperature was raised to 900 °C at a heating rate of 2 °C·min^−1^, maintain for 3 h, and then cool down naturally. After that, they were transferred into a muffle furnace and heated in air atmosphere. The temperature was raised to 400 °C at a rate of 2 °C·min^−1^ and maintained for 2 h, followed by natural cooling. Finally, Co/C and Fe/Co/C materials were obtained.

### 3.6. Crystallite Size Calculation

Through analysis of the XRD pattern, the crystal size can be obtained according to the Scherrer formula, as follows:

(1) $$D=\frac{K\lambda }{\beta \cos\theta }.$$

K stands for the constant, λ is the X-ray wavelength, β is half the height and width of the diffraction peak; and θ is the diffraction angle. In the above formula, the value of constant K is related to the definition of β—when β is half the width and height, K is 0.89; when β is the integral width, K is 1.0.

### 3.7. Dye Adsorption Experiment

For the dye removal experiment, the adsorption conditions were set as follows: the adsorbent dosage was 5 mg, the solution of CR was 10 mL (the concertation was 250 mg·L^−1^), the experimental temperature was 25 °C, the stirring speed was 150 rpm, and the adsorption time was set to 12 h. The concentration of the residual CR solution was analyzed at the maximum absorption wavelength of CR (λ_max_ = 497 nm) using a UV spectrophotometer. The concentration of the CR solution before and after adsorption was calculated according to the standard calibration curve (Figure S1). 

For the effect of the contact time experiment, samples were taken at 5, 10, 20, 30, 45, 60, 90, 120, 240, 480, and 720 min. The samples were centrifuged and detected using a ultraviolet spectrophotometer.

For the effect of initial concentration, the concentration of 50, 75, 100, 125, 150, 175, 200, 225, and 250 mg·L^−1^ was selected for testing. The samples were centrifuged and detected using a ultraviolet spectrophotometer.

To investigate the pH effect on the adsorption capacity of CR, adsorbents were added under different pH conditions. The pH was adjusted using HCl and NaOH solution (0.1 mol∙L^−1^). The pH was measured using a pH meter.

For the effect of the initial concentration after the addition of PMS, 250, 500, 750, and 1000 Congo red solutions were selected for testing, and the other conditions remained unchanged.

For the influence of the dosage of PMS, the ratio of Fe/Co/C to the dosage of PMS material was 1:0.5, 1:1, 1:2, and 1:3, and the other conditions remained unchanged.

For the cycle experiment, after the material was separated, it was washed with deionized water several times until the color of the deionized water did not change. The washed material was dried in an oven at 60 °C and then put into the next cycle experiment. 

### 3.8. Adsorption Isotherm and Kinetic Fitting

In this experiment, the adsorption capacity (q_e_) was calculated as follows:

(2) $$q_{e}=\frac{V\left(C_{0}-C_{e}\right)}{M}.$$

V stands for the volume of Congo red solution, C_0_ (mg·L−^1^) stands for the initial concentration of Congo red solution. and C_e_ (mg·L^−1^) stands for the Congo red concentration at equilibrium.

The adsorption isotherm is an effective method to analyze the adsorption process. Among the mathematical models, Langmuir, Freundlich, and Dubinin–Radushkevich adsorption isotherms are the most used [55,56]. In the Langmuir isotherm model, assuming that the adsorbents are adsorbed in only one layer, the adsorption sites are uniform and it has a reversible adsorption process [57]. Comparatively, the Freundlich adsorption isotherm is an empirical equation, it predicts that the surface of the adsorbent is heterogeneous, heterogeneous multilayer molecules are adsorbed, adsorbents have interactions, and the adsorption amount increases with increasing concentration [58]. The Dubinin–Radushkevich model is a model that can quantitatively describe the adsorption of gas and vapor via microporous adsorbents. This model is based on the assumption that the adsorption mechanism in micropores is pore filling, rather than layered surface coverage, and it is generally suitable for adsorption systems in which only van der Waals forces are involved [59].

The adsorption equilibrium isotherms were fitted via linear equations using the Langmuir model, Freundlich model, and Dubinin–Radushkevich model.

(3) $$\frac{\mathrm{Ce}}{\mathrm{Cq}}=\frac{1}{K_{L}q_{\max}}+\frac{\mathrm{Ce}}{q_{\max}}$$

K_L_ stands for the Langmuir equilibrium constant, which is related to the adsorption capacity; q_max_ (mg∙g^−1^) stands for the maximum monolayer adsorption capacity of the adsorbent; and Ce stands for the Congo red concentration at equilibrium.

(4) $$\ln q_{e}=\ln K_{F}+\frac{1}{n}\ln C_{e}$$

K_F_ stands for the Freundlich equilibrium constant and it is related to the adsorption capacity; 1/n stands for the adsorption strength.

(5) $$\ln q_{e}=\ln q_{max}-K_{D}\varepsilon^{2}$$

K_D_ stands for the Dubinin–Radushkevich equilibrium constant, ε stands for the adsorption potential.

Pseudo-first-order and pseudo-second-order models were used to fit the adsorption kinetics; their formulas are as follows:

(6) $$\mathrm{lg}\left(q_{e}-q_{t}\right)=\mathrm{lg}q_{e}-\frac{k_{1}t}{2.303},$$

(7) $$\frac{t}{q_{1}}=\frac{1}{k_{2}{q_{e}}^{2}}+\frac{t}{q_{e}}.$$

q_e_ (mg∙g^−1^) stands for the adsorption capacities at equilibrium time, q_t_ (mg∙g^−1^) stands for the adsorption capacities at actual time, k_1_ (min^−1^) is the rate constant of the pseudo-first-order kinetic model, and k_2_ (g∙mg^−1^·min^−1^) is the rate constant of pseudo-second-order kinetic model.

## 4. Conclusions

In summary, a Fe-doped, ZIF-67 derived magnetic composite material has been developed by employing chitosan via a facile strategy. Due to the high BET surface area and its abundant porous characteristics, the Fe/Co/C material exhibits a good adsorption effect for CR. The maximum adsorption capacity is 200.8 mg·g^−1^ and the adsorption equilibrium can be reached within 6 h. To further improve the removal capacity and efficiency, PMS was used to react with Fe/Co/C material due to its Fenton-like effect. Moreover, the material can be easily separated from water by applying a magnetic field because of its high saturation magnetization (85.4 emu·g^−1^). Thus, the problem that the adsorption materials are difficult to recover can be solved and secondary pollution can be avoided. After five cycles, the adsorption effect was still good. Furthermore, by studying the pH value, initial concentration, adsorption isotherm, and adsorption kinetics, the adsorption process and mechanism are proposed. This work illustrated a simple and flexible preparation of Fe-doped, ZIF-67 derived magnetic material; it can be doped with different metals to achieve specific targets. The study may provide possibilities for the development of functionalized MOF-based materials for dye treatment among printing and dyeing wastewater.

## Figures and Tables

**Table 1:** The proportion of surface elements obtained via XPS.

	O	C	Co	Fe
Co/C	24.54%	56.57%	19.69%	0
Fe/Co/C	27.8%	56.6%	4.79%	7.3%

**Table 2:** Adsorption equilibrium isotherm models of Fe/Co/C on CR.

Isotherm Models	Model Parameters	CR
Langmuir	q_m_	200.8
	K_L_	0.207
	R^2^	0.991
Freundlich	K_F_	37.8
	n	2.77
	R^2^	0.655
Dubinin–Raduskevich	lnq_m_	5.29
	K_D_	4.04
	R^2^	0.984

**Table 3:** The detail parameters of the pseudo-first-order and pseudo-second-order kinetic models for CR removal via Fe/Co/C.

Dye	C_0_ (mg·L^−1^)	qe, exp (mg·g^−1^)	Pseudo-First-Order Kinetic			Pseudo-Second-Order Kinetic		
			k_1_ (min^−1^)	R_1_^2^	qe_1_, cal (mg·g^−1^)	k_2_ (g∙mg^−1^·min^−1^)	R_2_^2^	qe_2_, cal (mg·g^−1^)
CR	250	201.6	0.01	0.952	201.37	3.87	0.960	239.8

## Data Availability

All data are available in the main text or the Supplementary Materials.

## Supplementary Materials

The following supporting information can be downloaded at: https://www.mdpi.com/article/10.3390/molecules29092078/s1, Figure S1. Standard curve of CR; Figure S2. Effects of different ZIF-67 content on CR treatment; Figure S3. Image of material after solution evaporation; Figure S4. Comparison of the adsorption capacity of Co/C and Fe/Co/C; Figure S5. (a) Adsorption/desorption curves of Fe/Co/C material and (b) pore size distribution of Fe/Co/C material; Figure S6. (a) Adsorption/desorption curves of Co/C material and (b) pore size distribution of Co/C material; Figure S7. FTIR patterns of ZIF-67 and Fe/Co/C; Figure S8. (a) Fitting of adsorption isotherms for linear Fe/Co/C Langmuir model. (b) Fitting of adsorption isotherms for linear Fe/Co/C Freundlich model. (c) Fitting of adsorption isotherms for linear Fe/Co/C Dubinin–Radushkevich model; Figure S9. (a) Pseudo-first-order kinetic fitting. (b) Pseudo-second-order kinetic fitting; Figure S10. Removal process changes image; Table S1. Comparison of other materials.
