# Supplementary material for: Fabrication of an Fe-Doped ZIF-67 Derived Magnetic Fe/Co/C Composite for Effective Removal of Congo Red
Source: Molecules. 2024 Apr 30;29(9):2078. doi: 10.3390/molecules29092078 (PMC11085441; doi:10.3390/molecules29092078)
Supplement: Supplementary file 1 [file molecules-29-02078-s001.zip › molecules-2938982-supplementary.pdf]

Electronic Supplementary information

Fabrication of a Fe-Doped ZIF-67 Derived Magnetic Fe/Co/C Composite for Effective Removal of Congo Red

Yu Cao [1,*], Zeming Dai [1], Xuan Zhou [1], Yuting Lin [1] and Jianhua Hou [1,2,*]

[1] College of Environmental Science and Engineering, Yangzhou University, Yangzhou 225000, China; dzmzeming@163.com (Z.D.); yzdxzx@yeah.net (X.Z.); Linyut@yeah.net (Y.L.)

[2] Jiangsu Collaborative Innovation Center for Solid Organic Waste Resource Utilization, Nanjing 210095, China

* Correspondence: yucao@yzu.edu.cn (Y.C.); jhhou@yzu.edu.cn (J.H.); Tel.: +86-514-8797868608 (Y.C.)

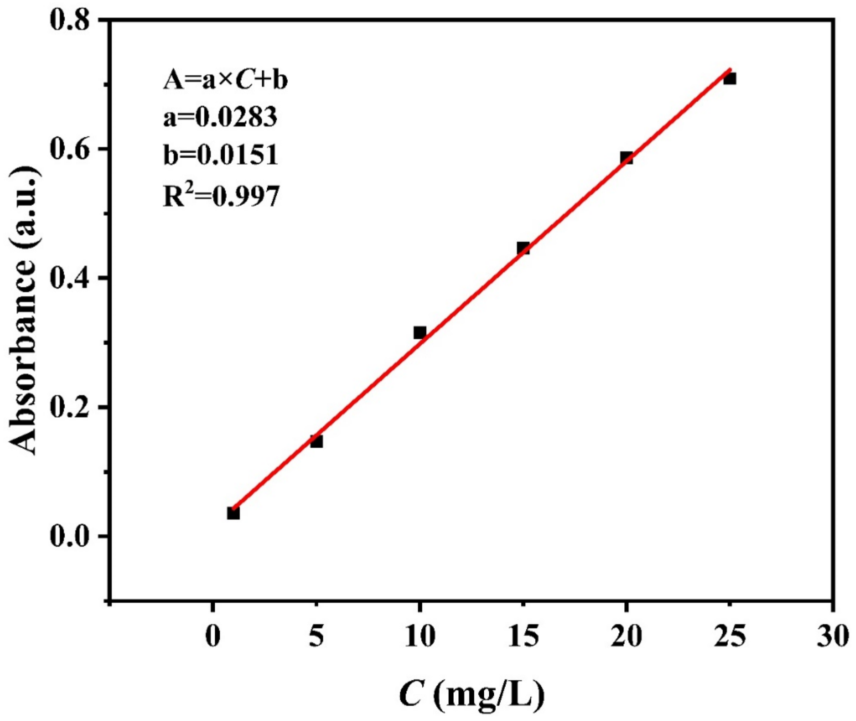

Figure S1. Standard curve of CR.

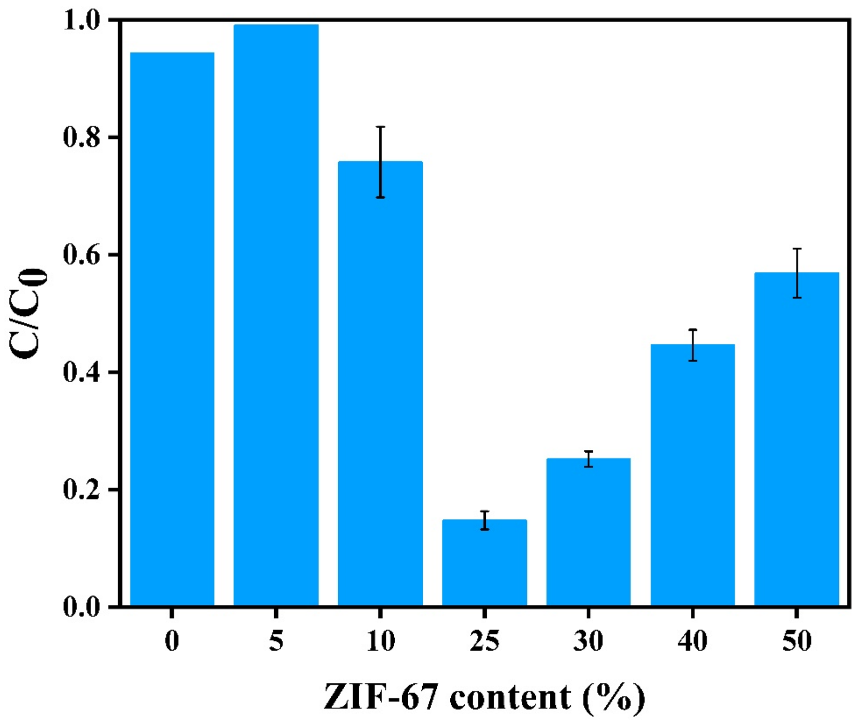

Figure S2. Effects of different ZIF-67 content on CR treatment.

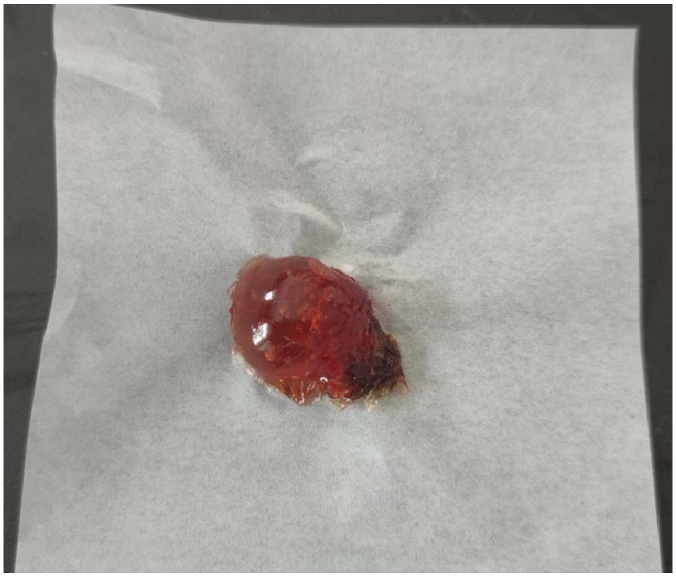

Figure S3. Image of material after solution evaporation.

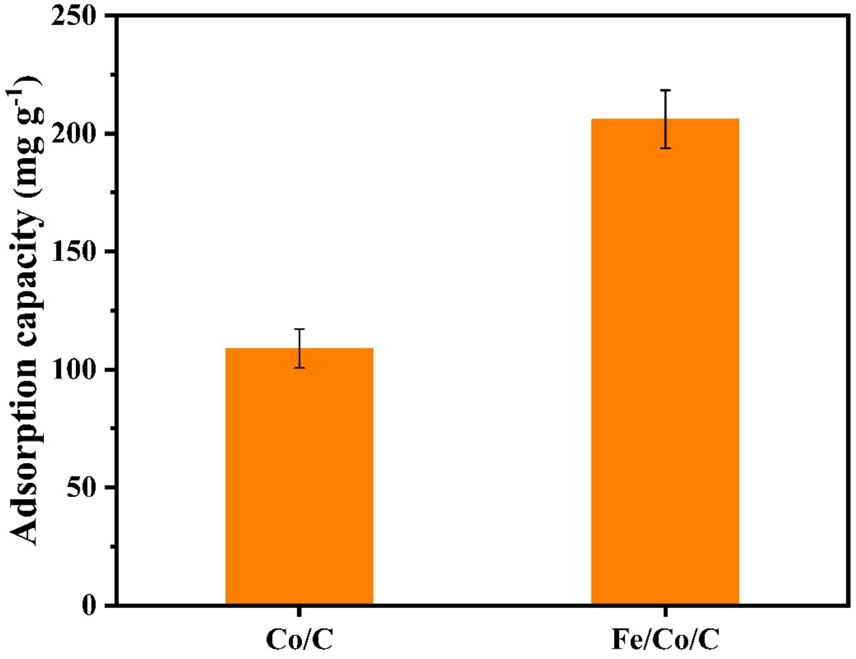

Figure S4. Comparison of the adsorption capacity of Co/C and Fe/Co/C.

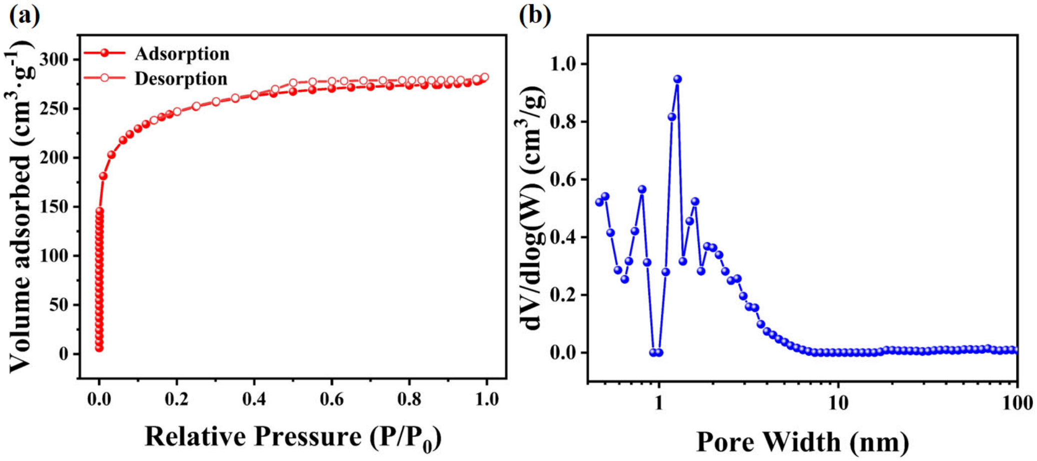

Figure S5. (a) Adsorption/desorption curves of Fe/Co/C material and (b) Pore size distribution of Fe/Co/C material.

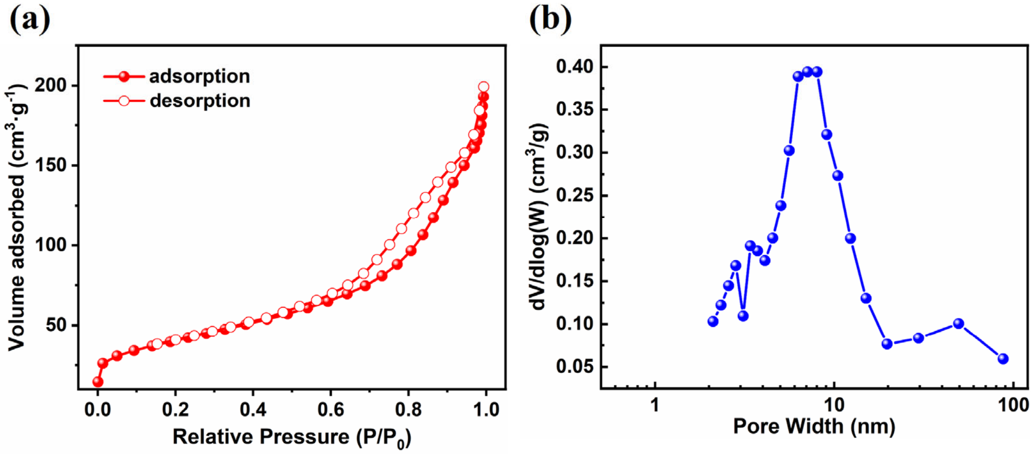

Figure S6. (a) Adsorption/desorption curves of Co/C material and (b) Pore size distribution of Co/C material.

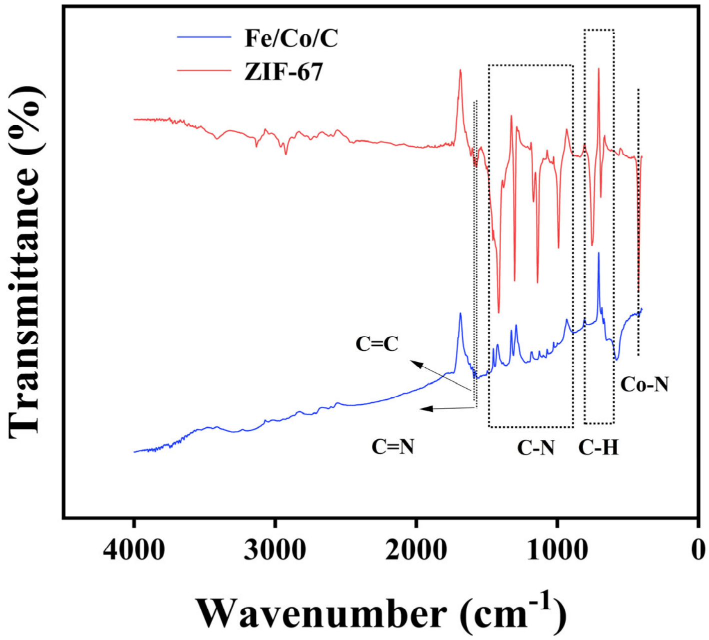

Figure S7. FTIR patterns of ZIF-67 and Fe/Co/C.

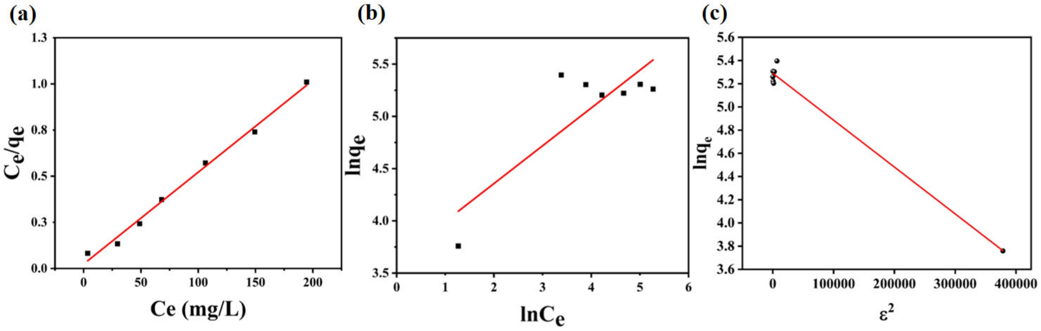

Figure S8. (a) Fitting of adsorption isotherms for linear Fe/Co/C Langmuir model. (b) Fitting of adsorption isotherms for linear Fe/Co/C Freundlich model. (c) Fitting of adsorption isotherms for linear Fe/Co/C Dubinin-Radushkevich model.

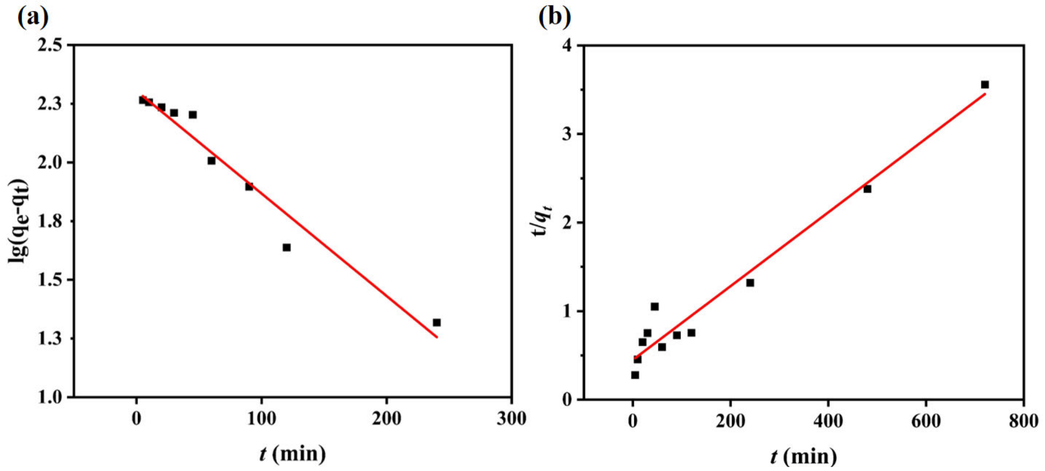

Figure S9. (a) Pseudo-first-order kinetic fitting. (b) Pseudo-second-order kinetic fitting.

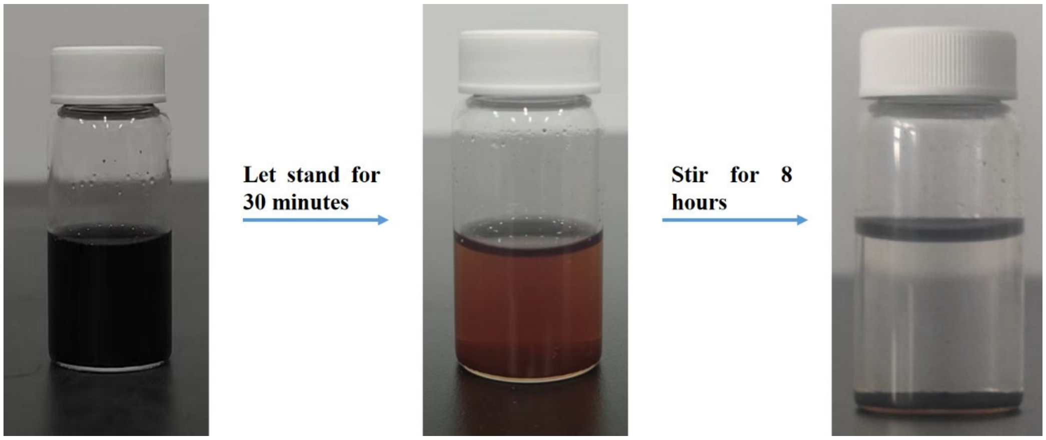

Figure S10. Removal process changes image.

Table S1. Comparison of other materials.

Name	Material classification	Saturation magnetization	Target pollution	q_m ($mg \cdot L^{-1}$)	Ref
Fe/Co/C	MOF-derived materials	84. emu g^{-1}	Congo red	200.8	**This work**
m-CS-c-PAM	composite material	5.74 emu g^{-1}	Sunset Yellow	359.71	48
MSPA	activated carbon	4.58 emu g^{-1}	Rhodamine B	475.49	49
nZVI/BC	composite material	38.7 emu g^{-1}	Malachite green	515.77	50
Gel-CNT-MNPs	composite material	31 emu g^{-1}	methylene blue	465.5	51
chitosan@Fe_3O_4	composite material	4.85 emu g^{-1}	Congo red	727.8	52
A. sphaerica-Fe_3O_4 (algalbased)	surface-modified Anabaena sphaerica biomass	2.76 emu g^{-1}	Remazol Red F3B	63.9	53

References (the serial number of the references corresponds to the order in the main article)

48. Jiang, R.; Shen, T.; Zhu, H.; Fu, Y.; Jiang, S.; Li, J.; Wang, J. Magnetic Fe3O4 embedded chitosan–crosslinked-polyacrylamide composites with enhanced removal of food dye: Characterization, adsorption and mechanism. *Int. J. Biol. Macromol.* **2023**, 227, 1234–1244.

49. Cheng, S.; Zhao, S.; Xing, B.; Shi, C.; Meng, W.; Zhang, C.; Bo, Z. Facile one-pot green synthesis of magnetic separation photocatalyst-adsorbent and its application. *J. Water Process Eng.* **2022**, 47, 102802.

50. Eltaweil, A.S.; Ali Mohamed, H.; Abd El-Monaem, E.M.; El-Subruiti, G.M. Mesoporous magnetic biochar composite for enhanced adsorption of malachite green dye: Characterization, adsorption kinetics, thermodynamics and isotherms. *Adv. Powder Technol.* **2020**, 31, 1253-1263.

51. Saber-Samandari, S.; Saber-Samandari, S.; Joneidi-Yekta, H.; Mohseni, M. Adsorption of anionic and cationic dyes from aqueous solution using gelatin-based magnetic nanocomposite beads comprising carboxylic acid functionalized carbon nanotube. *Chem. Eng. J.* **2017**, 308: 1133-1144.

52. Wang, H.; Luo, W.; Guo, R.; Li, D.; Xue, B. Effective adsorption of Congo red dye by magnetic chitosan prepared by solvent-free ball milling. *Mater. Chem. and Phys.* **2022**, 292, 126857.

53. Abdel-Wahed, M.S.; El-Kalliny, A.S.; Shehata, F.A.; El-Aty, A.M.A.; Gad-Allah, T. A one-pot green synthesis of magnetic adsorbent via anabaena sphaerica and its performance towards remazol red dye removal from aqueous media. *Chem. Eng. Sci.* **2023**, 279, 118939.
